# In-Plant Intervention Validation of a Novel Ozone Generation Technology (Bio-Safe) Compared to Lactic Acid in Variety Meats

**DOI:** 10.3390/foods10092106

**Published:** 2021-09-06

**Authors:** David A. Vargas, Diego E. Casas, Daniela R. Chávez-Velado, Reagan L. Jiménez, Gabriela K. Betancourt-Barszcz, Emile Randazzo, Dan Lynn, Alejandro Echeverry, Mindy M. Brashears, Marcos X. Sánchez-Plata, Markus F. Miller

**Affiliations:** 1 International Center for Food Industry Excellence, Department of Animal and Food Sciences, Texas Tech University, Lubbock, TX 79409, USA; andres.vargas@ttu.edu (D.A.V.); diego.casas@ttu.edu (D.E.C.); daniela.r.chavez@ttu.edu (D.R.C.-V.); reagan.brashears@ttu.edu (R.L.J.); gabbetan@ttu.edu (G.K.B.-B.); alejandro.echeverry@ttu.edu (A.E.); mindy.brashears@ttu.edu (M.M.B.); markus.miller@ttu.edu (M.F.M.); 2 Nebraska Beef Ltd., Omaha, NE 68107, USA; edazzo@nbeef.com; 3 BioSecurity Technology, Omaha, NE 68144, USA; dan@biosecuritytechnology.com

**Keywords:** ozone intervention, beef variety meats, lactic acid, microbial indicators

## Abstract

The objective of this experiment was to compare the antimicrobial efficacy of an aqueous ozone intervention and a lactic acid solution on natural microbiota of variety meats in a commercial beef processing plant. EZ-Reach™ swabs were used to collect 100 cm^2^ area samples before and after ozone and lactic acid intervention application for three different offals (head, heart, and liver). Each repetition included 54 samples per variety meat and antimicrobial for a total of 162 samples per repetition. Enumeration of total aerobic bacteria (APC) and *Escherichia coli* (EC) was performed on each sample. Microbial counts for both microorganisms evaluated were significantly reduced (*p* < 0.001) after lactic acid immersion (2–5%) and ozone intervention for all variety meats, with the exception of ozone intervention in EC counts of the heart samples. APC after lactic acid intervention was reduced on average by 1.73, 1.66, and 1.50 Log CFU/sample in the head, heart, and liver, respectively, while after ozone intervention, counts were reduced on average by 1.66, 0.52, and 1.20 Log CFU/sample. EC counts after lactic acid intervention were reduced on average by 0.96, 0.79, and 1.00 Log CFU/sample in the head, heart, and liver, respectively, while after ozone intervention, counts were reduced on average by 0.75, 0.62, and 1.25 Log CFU/sample. The aqueous ozone antimicrobial scheme proved to be a promising intervention for the in-plant reduction of indicator levels in variety meats, specifically heads, hearts, and livers.

## 1. Introduction

In 2019, the United States produced almost 27.16 billion pounds of beef, including variety meats, of which 14.1% were exported [1,2]. Edible bovine by-products, also called variety meats or offal, consist of edible internal organs such as head, heart, liver, and tripe, which can comprise up to 12% of the live weight of cattle, and which are an important source of beef protein for key consumer sectors in countries like Japan, China, Taiwan, Kenya, and Mexico [2]. In these countries, some variety meats are considered delicacies and are included as the main ingredient in some of the most important traditional dishes [3]. In countries like Japan, cow tongues are considered an expensive protein source, in Mexico dishes such as menudo (tripe soup) or lengua (tongue) use variety meats as main ingredients, while in other countries they are consumed as an inexpensive source of high-quality protein and nutrition [3]. While most offal is exported, many of the variety meats are incorporated into ground products, such as the chopped beef, ground beef, beef patties, hamburgers, and processed meats that are locally consumed in the United States. Indeed, heart is an important source of protein in processed meats due to its high functionality in sausage and canned meats. Moreover, novel ideas in the use of variety meats can be found in the literature, including beef tongue powder, which was developed as a multipurpose food ingredient with the use of a spray dryer with the claim that it retained both protein and fat content from the actual raw tongue [4]. Therefore, it is important that the edible offal used or incorporated into these types of products are treated with interventions that control the possible presence of pathogens to prevent illnesses and recalls due to contamination.

Multiple studies have characterized the microbial population of variety meats, suggesting the need for decontamination technologies [5,6,7]. Abd-El Malek et.al. [8] examined multiple edible bovine by-products (intestine, lung, rumen, head, heart, kidney, and liver) and determined the incidence of *Escherichia coli* O157:H7, *Salmonella enteritidis*, and *Salmonella typhimurium*. McLauchlin et al. [9] investigated the microbiological quality of liver pâté and observed that from 870 samples, 9% were unsatisfactory, with the most common causes being elevated counts of aerobic bacteria (6%), high Enterobacteriaceae counts (4%), or elevated counts of *Escherichia coli*, *Bacillus cereus*, and *Listeria monocytogenes*. Oblinger et al. [10] identified and isolated bacteria from fresh and temperature-abused variety meats (livers, kidneys, hearts, tongues, and pork livers) and from 1555 isolates obtained, *Micrococcus* spp. was the most common gram-positive bacterium in fresh variety meats, while *Escherichia coli* was the predominant gram-negative bacterium; in abused variety meats, *Pseudomonas* strains were predominant, except in vacuum packaging, where *Lactobacillus* spp. were predominately isolated. Few studies have evaluated the use of antimicrobials in order to decontaminate variety meats. Delmore et al. [11] examined the use of multiple interventions to reduce microbiological contamination of beef variety meats and determined that interventions already used to decontaminate beef carcasses can also be used for decontamination on variety meats, always considering an in-plant pre-validation of the intervention before relying on it in commercial settings. Also, the effectiveness of electrolyzed oxidizing water, hot water, and ozonated water was determined on bovine heads for the reduction of *Escherichia coli* O157:H7, resulting in optimistic results for controlling the foodborne pathogen [12].

BioSecurity Technology novel ozone intervention (Biosafe^TM^ cleaning solution) has been proven to decontaminate carcasses and trim in a commercial beef processing facility [13]. According to an in-plant study were carcasses and trim were intervened with ozone, a 1.29 Log CFU/cm^2^ and 0.67 Log CFU/500 cm^2^ reduction was observed in *Escherichia coli* counts for carcasses and trim, respectively [13]. Although contradictory results have been observed with ozone as an intervention on meat [14,15], novel ozone technologies based their differences on new ways to introduce ozone in an aqueous solution in order to avoid instability and reactivity before contact with the meat surface. Moreover, FSIS Directive 7120.1 asserts that ozone is safe for use in all meat products and no labeling requirements are needed, as it is considered a processing aid under the FDA definition, which specifies that a processing aid can be any substance that can be added to the food during processing but later it is either removed or its residual effects do not have any technical effect on the final product [16]. Meat by-products are normally discarded due to palate preferences in most countries [17,18]. The United States exports most organs, including hearts, tongues, livers, and heads, while blood and bones are incorporated as protein sources in animal feed or pet food. Variety meats are a high-quality source of protein, minerals, and vitamins, making them highly important products to help assure food security around the world [19].

Because increased levels of microbial populations and potential pathogens have been found in variety meats, and little information about interventions to reduce microbial contamination on variety meats is found in the literature on these highly important contributor products for food security, the objective of this study was to evaluate the efficacy of a novel ozone intervention on heads, hearts, and livers for reducing indicator microorganisms naturally present in variety meats in a commercial beef processing facility.

## 2. Materials and Methods

### 2.1. Intervention Parameters

Lactic acid intervention parameters included a spray intervention with a temperature of 43–55 °C at 2–4% lactic acid concentration, and spray pressure at ≥15 psi with 10 s contact time. Bio-safe ozone intervention parameters included proprietary ozone generators which utilize oxygen molecules (O_2_) split by a corona field, forming single atoms of oxygen (O_1_). These single atoms then are combined with O_2_ molecules forming a O_3_ molecule (ozone). The aqueous ozone intervention spray had a concentration 1.5–2.3 ppm, an oxidation-reduction potential (ORP) between 700 and 900 mV, spray pressure of ≥20 psi, and an incoming water source maintained at 10–24 °C. The ozone intervention consisted of one cabinet with 44 nozzles delivering 12.8 gpm of the solution with 18 s contact time. The plant processes around 2000 animals per day with just one shift from Monday to Saturday.

### 2.2. Variety Meat Samples

EZ-Reach^TM^ buffer peptone water (BPW) pre-hydrated 25 mL swabs (World Bioproducts, Mundelein, IL, USA) were taken using a 3M^TM^ cattle 100 cm^2^ template (3M, Saint Paul, MN, USA) before and after interventions in variety meats (head, heart, and liver). Each repetition included 54 samples (27 before and 27 after intervention) per variety meat for a total of 162 samples per repetition. A total of 6 repetitions were conducted throughout the experiment at different days and different times of the day in order to assure coverage and effect of time of the day and microbial accumulation in the processing facility, as well as to account for process variations that can be caused by operational and logistical issues in the plant.

### 2.3. Swab Processing

Pre-hydrated swabs were immediately chilled and shipped overnight to the ICFIE Food Microbiology Texas Tech University laboratory for microbiological analysis. Swab samples were homogenized in a stomacher (Model 400 circulator, Seward, West Sussex, UK) at 230 rpm for one minute. Then, samples were serially diluted in 9 mL BPW (Millipore Sigma, Danvers, MA, USA) tubes and plated to determine total aerobic plate counts (APC), and *Escherichia coli* counts (EC). This project was designed as a longitudinal experiment in which the beef processing plant changed their interventions as a program of continuous improvement at the processing line; therefore, the samples taken with lactic acid intervention were taken one year apart with the ozone samples. Samples taken before and after lactic acid intervention were enumerated using 3M^TM^ Petrifilm^TM^ (3M, Saint Paul, MN, USA) plates, while samples taken before and after ozone intervention were enumerated using the TEMPO system (BioMérieux, Paris, France) due to the separation in time at the moment of sampling both interventions. This study compares the microbial performance of year-to-year differences under modified antimicrobial schemes. For APC in Petrifilms, the Association of Official Agricultural Chemists 990.12 (AOAC) official method was used. Aerobic plate count petrifilms were incubated for 48 ± 3 h at 35 ± 1 °C. For *Escherichia coli*, the AOAC 991.14 official method was used. *Escherichia coli* petrifilms were incubated for 48 ± 3 h at 35 ± 1 °C. Enumeration was conducted at 48 h for *Escherichia coli* and APC. For APC and *Escherichia coli* in TEMPO^®^, the standard method was used, where TEMPO cards were incubated for 22–28 h at 35 ± 1 °C for both microorganisms following AOAC 121,204 for APC and AOAC 080,603 for *Escherichia coli*. This study also provides data to support the relationship between TEMPO^®^ and 3M^TM^ Petrifilm^TM^ enumeration technologies to support the validity of this study being conducted at different time periods and using different, but validated and comparable, microbial enumeration methods for the findings described in this study about the application of different interventions on variety meats in commercial processing facilities.

### 2.4. Tempo and Petrifilm Enumeration

A cocktail of five different strains of *Salmonella* (*Salmonella enterica* subsp. *enterica* ser. Typhimurium (BAA 712), *Salmonella enterica* subsp. *enterica* ser. Enteritidis (ATCC 31194), *Salmonella enterica* subsp. *enterica* ser. Infantis (BAA 1675), *Salmonella enterica* subsp. *enterica* ser. Newport (ATCC 6962), *Salmonella enterica* subsp. *enterica* ser. Senftenberg (ATCC 43845)) was used for quantification analysis. Microorganisms were obtained from the freezer where they were kept at −80 °C in a solution with 20% glycerol. Selected microorganisms were aseptically streaked for isolation on brain heart infusion (BHI) agar plates (Millipore Sigma, Danvers, MA, USA) and incubated for 24 h at 37 ± 1 °C. A single well-isolated colony from the BHI agar plate was regrown in another BHI plate and incubated for 24 h at 37 ± 1 °C. A single well-isolated colony of each serotype was suspended in 5 mL of sterile phosphate buffered saline water (PBS) (Sigma-Aldrich, MO, USA) and its concentration was verified using a Thermo Scientific™ Sensititre™ Nephelometer (Thermo fisher, MA, USA), calibrated to 0.5 McFarland turbidity, to obtain a concentration of 1~2 × 10^8^ CFU/mL. All the tubes with the different strains were mixed in a 15 mL conical tube to create the cocktail. The cocktail was serially diluted in buffer peptone water (BPW) tubes (Millipore Sigma, Danvers, MA, USA). Eight tubes containing a concentration ranging from 1 × 10^1^ to 1 × 10^8^ were treated as independent samples. Each tube was diluted for testing the measurements by the commercial procedures of bacterial enumeration of: (1) Direct plating using drop plating and micro dilution on Trypic Soy Agar (TSA) plates (Millipore Sigma, Danvers, MA, USA), (2) APC 3M^TM^ Petrifilm^TM^, (3) and most probable number (MPN) using TEMPO^®^ system AC. Four repetitions were conducted throughout the whole study.

(1) Direct plating. For each independent tube, 10-fold dilution in buffered peptone water was performed, 10 and 100 µL for drop and spread plating, respectively, were plated on TSA according to each dilution. After the drops were absorbed on the agar, plates were incubated and enumerated after 17–20 h at 35 ± 1 °C.
(2) 3M™ Petrifilm™. For APC in Petrifilms, the Association of Official Agricultural Chemists 990.12 (AOAC) official method was used. Aerobic plate count petrifilms were incubated for 48 ± 3 h at 35 ± 1 °C.
(3) TEMPO^®^. The Biomérieux TEMPO^®^ system was utilized to enumerate total counts of AC. For this method, samples were diluted based on the expected counts of each respective organism. This dilution was formulated within the provided TEMPO^®^ media vials and then loaded into a TEMPO^®^ card, which mimics a traditional 16 tube MPN method. These cards were then incubated for 22–28 h at 35 ± 1 °C following AOAC 121,204 for APC. After incubation, the cards were inserted into the TEMPO^®^ reader and enumerative values were calculated by the system.

### 2.5. Statistical Analysis

All data were analyzed using R (Version 4.0.4) statistical analysis software to evaluate the reduction of microbial loads after each intervention for all variety meats tested (head, heart, and liver). Counts were transformed into Log CFU/sample and a one-way ANOVA was performed, comparing before and after counts on each of the different interventions in the plant, followed by pairwise multiple comparison *T*-tests adjusted by the Benjamin and Hochber method. If parametric assumptions were not met, the Kruskal–Wallis Test was used as a non-parametric alternative for the ANOVA, followed by a pairwise multiple comparison Wilcoxon’s Test adjusted by the Benjamin and Hochber method. A *p*-value of 0.05 or less was selected prior to the analysis to determine significant differences.

For the 3M™ Petrifilm™ and TEMPO^®^ correlation experiments, all data were analyzed using R (Version 4.04) statistical analysis software to evaluate the relationship between the use of the two alternative enumeration technologies with the standard method. A linear model was calculated for each alternative enumeration method (3M™ Petrifilm™ and TEMPO^®^) where log_10_ counts from each of the alternative enumeration methods were considered as the dependent variable, while log_10_ counts obtained from direct plating (standard method) were considered as the independent variable.

## 3. Results

**Figure 1:**
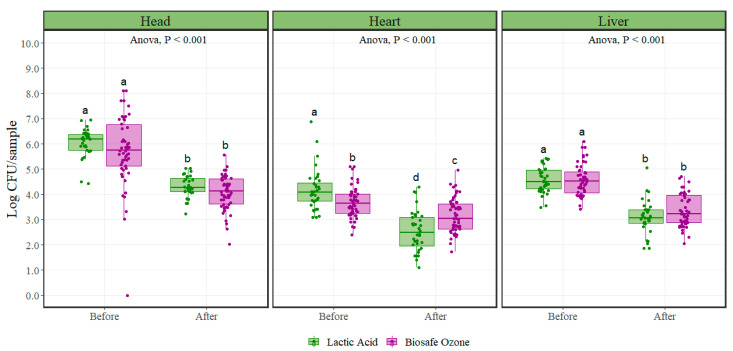
Mesophilic aerobic plate counts (Log CFU/sample; equivalent to Log CFU/100 cm^2^) before and after lactic acid and ozone interventions on variety meats (n = 54 per treatment). In each boxplot, the horizontal line crossing the box represents the median, the bottom and top of the box are the lower and upper quartiles, the vertical top line represents 1.5 times the interquartile range, and the vertical bottom line represents 1.5 times the lower interquartile range. ^a–d^ For each individual variety meat, boxes with different letters are significantly different according to ANOVA analysis followed by a pairwise comparison *T*-test at *p* < 0.05. The points represent the actual data points.

**Figure 2:**
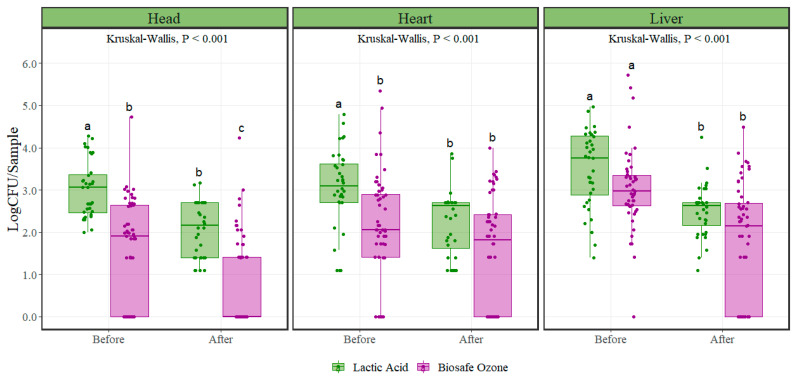
*Escherichia coli* counts (Log CFU/sample; equivalent to Log CFU/100 cm^2^) before and after lactic acid and ozone intervention on variety meats (n = 54 per treatment). In each boxplot, the horizontal line crossing the box represents the median, the bottom and top of the box are the lower and upper quartiles, the vertical top line represents 1.5 times the interquartile range, and the vertical bottom line represents 1.5 times the lower interquartile range. ^a–c^ For each individual variety meat, boxes with different letters are significantly different according to Kruskal–Wallis analysis followed by pairwise comparison Wilcoxon’s test at *p* < 0.05. The points represent the actual data points.

Aerobic plate counts (Figure 1) and *Escherichia coli* counts (Figure 2) were transformed to Log CFU/sample for statistical analysis, as counts were considerably low when analyzed on a Log CFU/cm^2^ basis (detection limit = 0.002 CFU/cm^2^). For all variety meats, both aerobic plate counts, and *Escherichia coli* counts were below 1 CFU/cm^2^, resulting in negative values when transformed to Log CFU/cm^2^, thus making analysis and visualization problematic and hard to interpret. Counts reported by Log CFU/sample were achieved by multiplying the CFU/cm^2^ counts by 100 cm^2^ of area sampled and then log_10_ converted, resulting in Log CFU/100 cm^2^ measurements, which were equivalent to Log CFU/sample.

Both the lactic acid and ozone interventions significantly reduced (*p* < 0.001) aerobic plate counts when applied to variety meats (Figure 1), (Table 1). Aerobic plate counts after lactic acid intervention were significantly reduced on average by 1.73, 1.66, and 1.50 Log CFU/sample in the head, heart, and liver, respectively. Likewise, samples collected under the ozone intervention scheme showed counts significantly reduced on average by 1.66, 0.52, and 1.20 Log CFU/sample in the head, heart, and liver, respectively. In the head and liver variety meats, for aerobic plate counts, there were no significant differences (*p* = 0.98) in microbial initial populations between the lactic acid and ozone interventions. Heart microbial initial populations were significantly different (*p* = 0.006) between the lactic acid and ozone interventions.

For *Escherichia coli* counts, a non-parametric approach test was used for analysis as these types of tests do not assume any specific distribution, as parametric tests do. The distribution of the data, especially after intervention, suggests a non-normal distribution due to the consistently observed lower counts. When assumptions were not met for performing ANOVA, a Kruskal–Wallis test was preferred to find differences between interventions. Both the lactic acid and the ozone interventions significantly reduced (*p* < 0.001) *Escherichia coli* counts in variety meats, with the exception of ozone intervention in heart samples (Figure 2) (Table 2). *Escherichia coli* counts after lactic acid intervention were significantly reduced on average by 0.96, 0.79, and 1.00 Log CFU/sample in the head, heart, and liver, respectively. Likewise, *Escherichia coli* counts after ozone intervention were reduced on average by 0.75, 0.62, and 1.25 Log CFU/sample in the head, heart, and liver, respectively. In head and heart samples, *Escherichia coli* microbial initial populations were significantly different between the lactic acid and ozone interventions (*p* < 0.001). No difference was observed (*p* = 0.15) in microbial populations between the two interventions in the liver samples.

**Figure 3:**
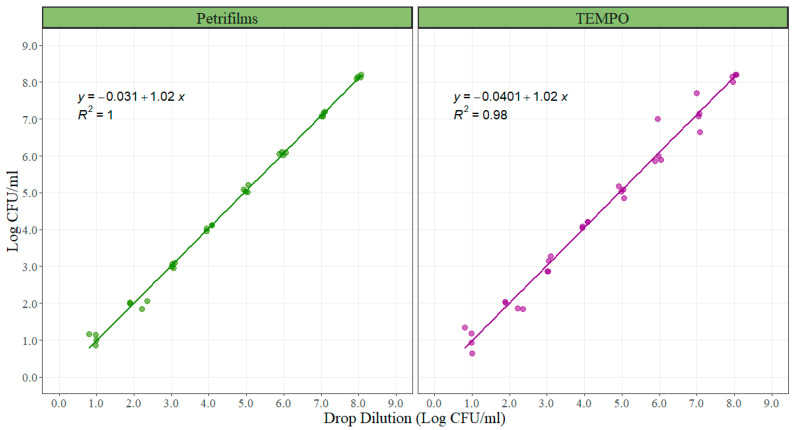
Graphic representation of the linear correlation between the bacterial counts of the alternative methods (3M™ Petrifilm™ and TEMPO^®^ system) when compared with the standard method (direct plating), (n = 32 per method).

For the correlation experiment between 3M™ Petrifilm™ and TEMPO^®^ with the standard method (direct plating), counts were log_10_ transformed and then analyzed. A linear model was created comparing each of the alternative enumeration methods with the standard method. The slope of the linear models represents the rate of change in bacterial counts in the alternative methods due to an increment of 1 unit in the standard method. It is ideally desired that the slope is one, meaning that for each 1 Log CFU/mL of increase in one of the alternative methods the standard method will increase 1 Log CFU/mL, making them comparable. The intercept represents the similarity in the total magnitude read by the compared methods. The slopes for both alternative enumeration methods when compared with direct plating were 1.02 with an adjusted r-squared of 1.00 for the 3M™ Petrifilm™ and 0.98 for the TEMPO^®^ system (Figure 3). The intercept value was −0.031 for the 3M™ Petrifilm™ and −0.0401 for the TEMPO^®^ system. Both intercepts were not statistically different from zero (*p* = 0.555 and *p* = 739), (Table 3).

## 4. Discussion

Aqueous ozone has been previously used as an antimicrobial intervention in beef. In a study where aqueous ozone was used as a spray chill intervention to reduce bacteria on surfaces of fresh beef, a significant reduction of 0.99 and 1.46 Log CFU/cm^2^ was achieved in aerobic bacteria and *Escherichia coli* O157:H7, respectively [20]. Moreover, in an in-plant validation study where aqueous ozone was used as an intervention on beef carcasses and trim, aerobic bacteria were reduced 3.26 Log CFU/cm^2^ and 0.74 Log CFU/500 cm^2^ for both carcasses and trim. Also, a reduction of 1.29 Log CFU/cm^2^ and 0.67 Log CFU/500 cm^2^ was reported for *Escherichia coli* in both carcasses and trim with a 75.5% reduction in the presence of presumptive *Escherichia coli* O157:H7 positives over a 1-year period [13]. For both studies, different but enhanced ozone technologies were used, as in this experiment, in order to increase the reactivity of ozone in an aqueous solution such that it may increase the efficacy of ozone intervention in beef.

Multiple experiments have observed significant reductions on microbial populations in meat samples [21,22,23]. In these studies, the results are extended to conclusions about beef variety meats. Aqueous ozone interventions have not been extensively reported in variety meats; however, in this experiment, there was a reduction of 1.73, 1.66, and 1.50 Log CFU/sample in the head, heart, and liver for aerobic bacteria and of 0.75, 0.62, and 1.25 Log CFU/sample in *Escherichia coli*. When comparing the reduction obtained by ozone intervention against lactic acid intervention, the values were equivalent in magnitude in this experiment, as well as in other studies where reductions in variety meats were between 0.7 to 1.7 Log CFU/g for aerobic bacteria and 0.1 to 1.1 Log CFU/g for *Escherichia coli* [11]. Lactic acid interventions are known for not only having an immediate reduction on the microbial populations of bacteria, but also for their potential residual effect in the reduction of microbial loads [24]. By contrast, ozone has no attributed residual effect due to high reactivity; furthermore, it is known to be unstable and to react with organic materials, easily suggesting an increased immediate reduction of microbial populations of bacteria in variety meats. Shelf-life studies should be conducted in order to assess the possible differences between both interventions over longer periods of storage time for heads, hearts, and livers, as well as the use of surrogates that simulate the survival of pathogens in an in-plant environment, thus testing the real efficacy of an intervention during meat processing in a commercial processing facility.

Since the implementation of the HACCP rule in 1996, the U.S beef packaging industry started to investigate and implement multiple interventions intended to reduce the microbial population on carcasses and to reduce the presence of foodborne pathogens on sub-primal cuts and trimmings [25]. Limited information about performance standards on variety meats are found in the U.S., however, variety meat processors also need to comply with food safety requirements, as these meats can be incorporated into ground beef. A comprehensive literature search for guidelines outside the U.S. only returned limited publications on microbiological criteria for hearts, heads, and livers by the Kenyan Bureau of Standards and the Gulf Cooperation Countries Standardization Organization standards, establishing a limit for aerobic colony counts of 6 Log CFU/g, 2 Log CFU/g for *Escherichia coli*, and *Salmonella* absence [26,27] in this type of offal. In this experiment, on average, *Escherichia coli* counts were below 1 CFU/cm^2^ after intervention with lactic acid or ozone, thus the facility can demonstrate appropriate process control while using lactic acid or ozone on most of the variety meats evaluated in this experiment. Also, it is worth mentioning that these three tested variety meats were chosen by the plant to be tested as a worst-case scenario. The plant used historical data to determine that these three variety meats were those with greater microbial loads following their slaughter process, and the study was designed to support continuous improvement process control.

Ozone is well known to be an effective antimicrobial agent with high reactivity, penetrability, and decomposition to a non-toxic product [28]. Its mechanism of disinfection includes direct oxidation and lysis of the cell wall, reactions with radical by-product of ozone decomposition and possible DNA damage (nucleic acids) [29]. Ozone applications in the food industry are mostly related to disinfection of surfaces, water treatment, and inactivation of contaminant flora on vegetables and dry foods. For many years, studies of ozone interventions on meat and poultry have been contradictory, with the results obtained being project related and dependent on the delivery mechanisms of active ozone to the treated surfaces. However, novel technologies to incorporate highly reactive ozone in an aqueous solution are giving promising results for the meat and poultry industry, which has sought alternative interventions for decades.

As mentioned above, this project was designed as a longitudinal experiment where the beef processing plant changed their interventions as a program of continuous improvement at the processing line. Because of this and the space in between the sampling points, different, but standardized, enumeration technologies were used in order to test the effects of lactic acid and ozone in variety meats. In order to support the accuracy of the results of this experiment, a correlation experiment between the quantifications using 3M™ Petrifilm™ and TEMPO^®^ in comparison with the standard method (direct plating) was performed. The estimated slopes for both alternative enumeration methods were 1.02 Log CFU/mL, suggesting that for 1 Log CFU/mL increase in counts using the standard method, a 1.02 Log CFU/mL increase in counts will be obtained using both alternative quantification methods. The 95% confidence interval of the slopes contain the value of 1 for both enumeration methods, which means that the slope values were not statistically different from 1, suggesting that the differences observed were just due to normal experimental error (Table 2). The similarity in the total magnitude read by the methods is an important parameter that is represented by the intercept of the linear models. The estimated intercepts for both alternative enumeration methods were not statistically different from zero, suggesting that the magnitudes of both methods were similar when compared to the standard method (direct plating).

The significance of this study lies in the importance of using validated decontamination technologies on variety meats due the natural microbiota found in these meat products, as well as their value in some countries as a low-cost source of protein that contributes to food security. Furthermore, little information can be found on the literature about in-plant validation studies for the decontamination of variety meats. The importance of testing decontamination technologies in commercial beef processing facilities should be imperative as the efficacy of a decontamination technology can be addressed in real conditions, thus giving reliable results. Moreover, the limited information found about baseline microbial data on variety meats and regulations or performance standards associated with either the variety meats themselves or their production is something that should be considered, as most of these products are added as functional ingredients to processed meats, ground to produce ground beef or exported to countries where they are consumed as delicacies or as an important source of protein.

## 5. Conclusions

The aqueous ozone antimicrobial scheme proved to be promising as an intervention for the reduction of microbial indicator levels in variety meats. These findings suggest that bio-safe ozone intervention could play a competitive role with equivalent reductions obtained with lactic acid applications on reducing bacterial loads on variety meats, thus contributing to food quality, and potentially be used as safety control tool. The validation of the technology in a commercial beef processing plant gives additional support for feasibility and effectiveness of the technology on reducing microbial counts on variety meats.

## Figures and Tables

**Table 1:** Summary table of aerobic plate counts in all variety meats before and after lactic acid and biosafe ozone interventions.

Variety Meat	Intervention	Aerobic Plate Counts (Log CFU/Sample ^1^ ± SE ^2^)		*p*-Value
		Before	After	
Head	Lactic Acid	6.04 ± 0.10 ^a^	4.31 ± 0.07 ^b^	<0.001
	Biosafe Ozone	5.75 ± 0.19 ^a^	4.09 ± 0.09 ^b^	
Heart	Lactic Acid	4.19 ± 0.13 ^a^	2.53 ± 0.14 ^d^	<0.001
	Biosafe Ozone	3.68 ± 0.08 ^b^	3.16 ± 0.10 ^c^	
Liver	Lactic Acid	4.57 ± 0.08 ^a^	3.07 ± 0.11 ^b^	<0.001
	Biosafe Ozone	4.57 ± 0.09 ^a^	3.37 ± 0.09 ^b^	

^1^ Log CFU/sample is equivalent to Log CFU/100 cm^2^; ^2^ Standard error of the mean; ^a–d^ For each individual variety meat, values with different letters are significantly different according to Kruskal–Wallis analysis followed by pairwise comparison Wilcoxon’s test at *p* < 0.05.

**Table 2:** Summary table of *Escherichia coli* counts in all variety meats before and after lactic acid and biosafe ozone interventions.

Variety Meat	Intervention	*Escherichia coli* (Log CFU/Sample ^1^ ± SE ^2^)		*p*-Value
		Before	After	
Head	Lactic Acid	3.02 ± 0.11 ^a^	2.06 ± 0.11 ^b^	<0.001
	Biosafe Ozone	1.55 ± 0.17 ^b^	0.80 ± 0.15 ^c^	
Heart	Lactic Acid	3.04 ± 0.16 ^a^	2.25 ± 0.12 ^b^	<0.001
	Biosafe Ozone	2.12 ± 0.18 ^b^	1.50 ± 0.18 ^b^	
Liver	Lactic Acid	3.50 ± 0.15 ^a^	2.50 ± 0.10 ^b^	<0.001
	Biosafe Ozone	3.03 ± 0.14 ^a^	1.78 ± 0.19 ^b^	

^1^ Log CFU/sample is equivalent to Log CFU/100 cm^2^; ^2^ Standard error of the mean; ^a,b^ For each individual variety meat, values with different letters are significantly different according to Kruskal–Wallis analysis followed by pairwise comparison Wilcoxon’s test at *p* < 0.05.

**Table 3:** Summary table of linear models using the least squares regression method predicting the bacterial counts of 3M™ Petrifilm™ and TEMPO^®^ when compared with with the standard method (direct plating).

Enumeration Method	Coefficient	Estimate	Standard Error	*p*-Value	95% Confidence Intervals	
					Lower (2.5%)	Upper (97.5%)
TEMPO^®^	Intercept	−0.040	0.119	0.739	−0.273	0.193
	Slope	1.023	0.024	<0.001	0.976	1.070
3M™ Petrifilm™	Intercept	−0.031	0.052	0.555	−0.133	0.071
	Slope	1.018	0.010	<0.001	0.998	1.038

## Data Availability

Data available on request from the corresponding author. The data are not publicly available due to privacy from the beef processing partner that allowed the project to be conducted within their beef processing environment.
